# Bark-dwelling methanotrophic bacteria decrease methane emissions from trees

**DOI:** 10.1038/s41467-021-22333-7

**Published:** 2021-04-09

**Authors:** Luke C. Jeffrey, Damien T. Maher, Eleonora Chiri, Pok Man Leung, Philipp A. Nauer, Stefan K. Arndt, Douglas R. Tait, Chris Greening, Scott G. Johnston

**Affiliations:** 1 Southern Cross Geoscience, Southern Cross University, Lismore, NSW Australia; 2 Faculty of Science and Engineering, Southern Cross University, Lismore, NSW Australia; 3 Department of Microbiology, Biomedicine Discovery Institute, Monash University, Clayton, VIC Australia; 4 School of Chemistry, Monash University, Clayton, VIC Australia; 5 School of Ecosystem and Forest Sciences, University of Melbourne, Richmond, VIC Australia

**Keywords:** Bacterial evolution, Plant ecology, Carbon cycle, Carbon cycle

## Abstract

Tree stems are an important and unconstrained source of methane, yet it is uncertain whether internal microbial controls (i.e. methanotrophy) within tree bark may reduce methane emissions. Here we demonstrate that unique microbial communities dominated by methane-oxidising bacteria (MOB) dwell within bark of *Melaleuca quinquenervia*, a common, invasive and globally distributed lowland species. In laboratory incubations, methane-inoculated *M. quinquenervia* bark mediated methane consumption (up to 96.3 µmol m^−2^ bark d^−1^) and reveal distinct isotopic δ^13^C-CH_4_ enrichment characteristic of MOB. Molecular analysis indicates unique microbial communities reside within the bark, with MOB primarily from the genus *Methylomonas* comprising up to 25 % of the total microbial community. Methanotroph abundance was linearly correlated to methane uptake rates (R^2^ = 0.76, *p* = 0.006). Finally, field-based methane oxidation inhibition experiments demonstrate that bark-dwelling MOB reduce methane emissions by 36 ± 5 %. These multiple complementary lines of evidence indicate that bark-dwelling MOB represent a potentially significant methane sink, and an important frontier for further research.

## Abstract (web summary)

The photosynthesis performed by trees makes them an important sink for atmospheric carbon dioxide, but trees are also sources of the potent greenhouse gas methane. Here the authors find that tree bark in some common lowland species is colonized by methane oxidizing bacteria that can reduce tree methane emissions by ~ 36%.

## Introduction

Methane (CH_4_) is ~32 to 87 times more potent than carbon dioxide at warming the Earth’s atmosphere^1^ Methane emissions from tree stems has received growing attention and is considered a new frontier in the global carbon cycle^2–6^. With an estimated three trillion trees on Earth^7^ and reforestation/afforestation promoted as viable climate change mitigation strategies^8–11^, a mechanistic understanding of the processes driving and moderating methane emission from trees is of critical importance. Freshwater wetland trees typically emit much higher rates of methane^5,12,13^ than their mangrove^14^ and upland forest counterparts^15–19^. This is because the poorly drained, carbon-rich soils typical of freshwater wetland forests are favourable for methanogenesis. Recent research revealed that lowland trees contributed ~50% of the Amazon methane emission budget^5^, highlighting the potential importance of this emission pathway. However, a lack of data on tree-mediated methane emissions has prevented their inclusion in global methane budgets^4^.

Methane-oxidising bacteria (MOB) can decrease methane emissions in a wide range of natural environments. Wetlands are recognised as Earth’s largest natural source of atmospheric methane^4,20^, yet 50-90% of the methane produced within wetlands may be oxidised before reaching the atmosphere^21–23^. Although the importance of MOB within wetland soil and water is well documented^23–28^, their possible role within trees has yet to be characterised. Methanogenic archaea have been identified within the heartwood and sapwood of several lowland tree species^29–32^, but the operational taxonomic units of methanotrophic families were exceedingly rare^30^ and their influence on tree stem methane emissions remains unquantified. Until now, it is unclear if bark may provide a habitat for MOB.

Here, we establish that tree stem bark can host previously uncharacterised microbiomes and unique MOB communities that substantially mitigate tree stem methane emissions and thereby help regulate Earth’s climate. Our study combined the use carbon stable isotope analysis^24,28,33,34^, in situ methanotrophy inhibitors^35,36^ and molecular community profiling^37,38^, which have each been previously used to determine the rates and mediators of microbial oxidation in wetlands and other environments. On this basis, we provide multiple lines of biogeochemical and microbial evidence that abundant MOB occupy tree bark and represent an uncharacterised methane sink.

## Results and discussion

### Methane oxidation potential and fractionation during bark incubations

**Fig. 1:**
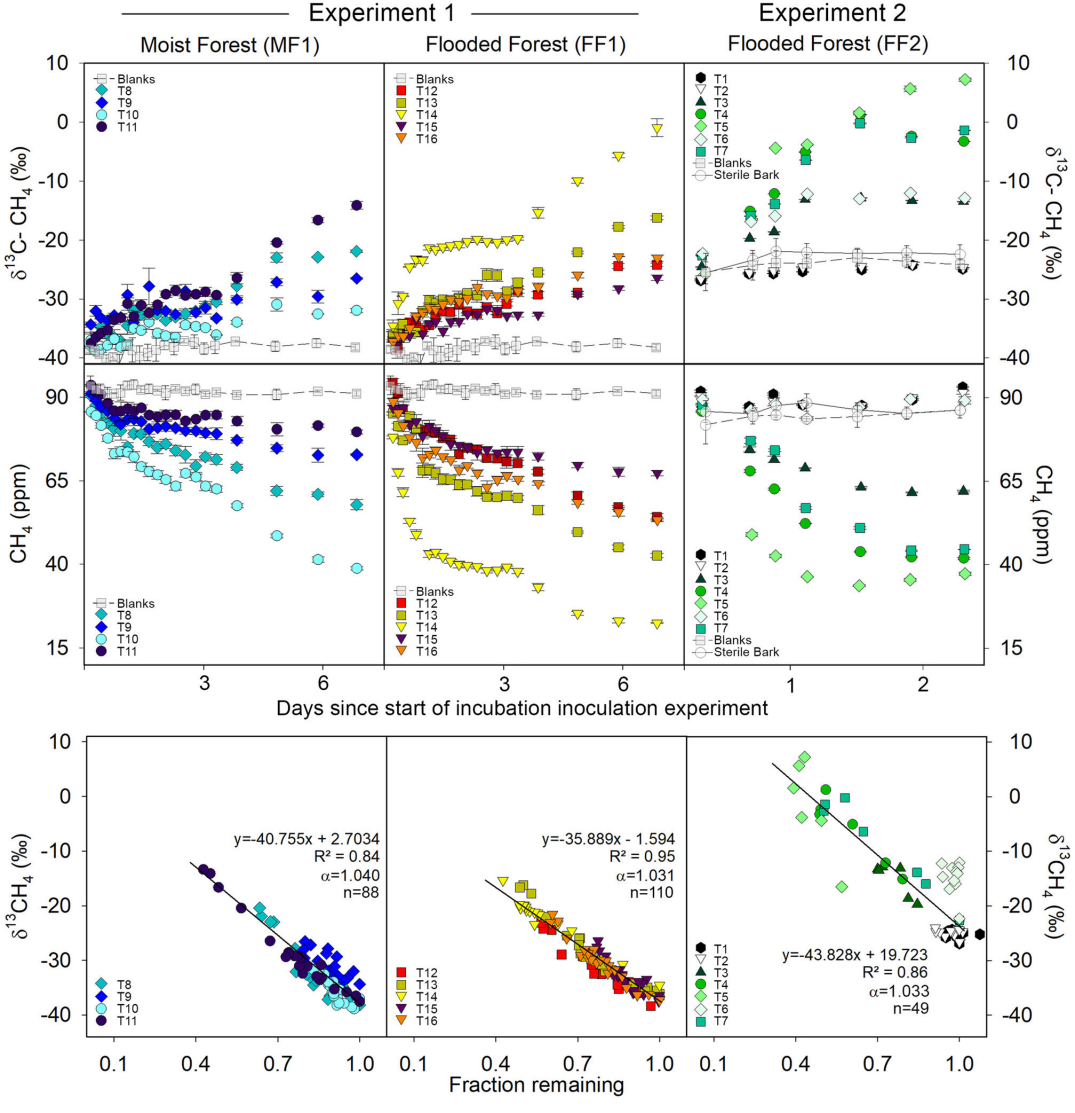
Methane oxidising bacteria (MOB) time series incubation experiments of methane-inoculated *M. quinquenervia* bark. The panels depict oxidation as δ^13^C–CH_4_ enrichment vs time (top), decrease in methane concentration (ppm) vs time (middle) and the δ^13^C–CH_4_ vs fraction remaining (bottom). Note: Different δ^13^C–CH_4_ (‰) starting values between the first (MF, FF1) and second (FF2) experiments are due to using a different methane gas standard. Coloured symbols represent each bark sample (see Supplementary Table 1, T = tree), error bars are ±SD and α = fractionation factor. Average values for both controls (blank bottles and sterilised bark) are shown as grey symbols with trend line. Note: T1, T2 and T6 were removed from fraction remaining correlation due to lack of MOB oxidation, which was supported by lower MOB abundance within the paired bark samples (see microbial data in Fig. 2). Fraction remaining is the proportion of methane not oxidised by MOB during the time series.

**Fig. 2:**
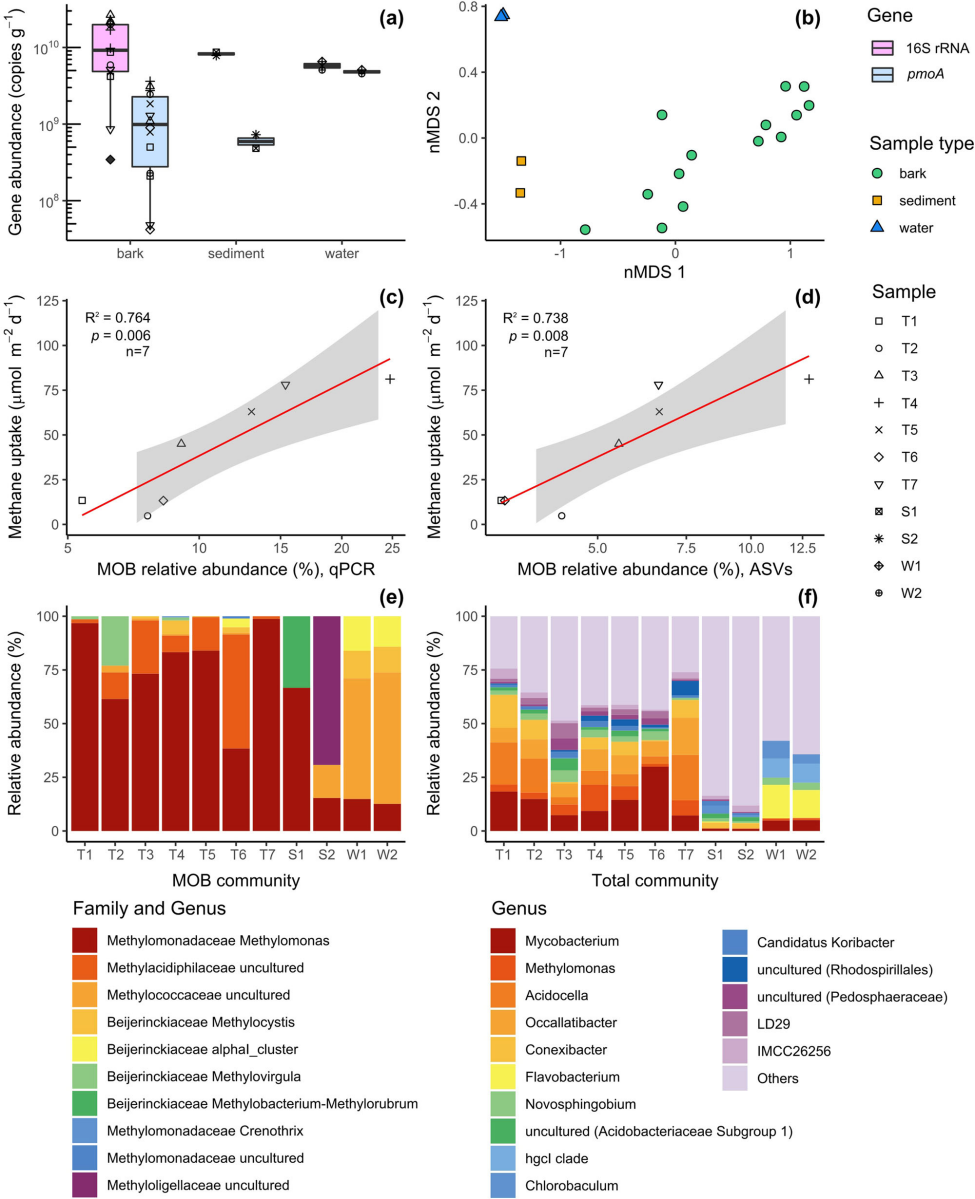
Summary of abundance, composition and structure of total microbial and methanotroph (MOB) communities in *M. quinquenervia* bark (*n* = 14, T = tree), sediment sample (*n* = 2, S = sediment) and water samples (*n* = 2, W = water). **a** Abundance determined by quantitative PCR of the total microbial community (universal 16 S rRNA gene copy number) and of the MOB community (*pmoA* gene copy number). Box plots depict medians, lower and upper quartiles and maximum and minimum values. **b** Non-metric multidimensional scaling (nMDS) ordination of the MOB community structure (beta diversity) measured by Bray–Curtis distance matrix of the 16 S rRNA gene amplicon sequences affiliated with known methanotrophic families and genera. **c**, **d** Correlation between laboratory incubation measurements of the first 24 h of methane uptake from bark samples (Supplementary Table 1) and logit-transformed MOB community proportion in the total community (percentage of MOB relative abundance) inferred from qPCR (**c**) and 16 S rRNA amplicon sequence variants (**d**) (linear regression and *t* test; *n* = 7; df = 6; the grey area indicates 95% confidence interval) **e** Relative abundance of methanotrophic genera identified from the analysis of the 16 S rRNA gene amplicon sequences. In the case of uncultured genera, taxonomic resolution according to family is reported. **f** Relative abundance of 16 S rRNA gene amplicon sequences resolved at the taxonomic level of genus.

In order to detect MOB activity, we monitored methane concentrations and isotope fractionation in methane-inoculated gas-tight bottles containing freshly collected *Melaleuca quinquenervia* bark samples from three different sites (see Supplementary Methods). Because the heavier ^13^C–CH_4_ isotope contains slightly stronger bonds, MOB preferentially consume ^12^C–CH_4_, thereby triggering isotopic fractionation. Two laboratory time series experiments both revealed clear methane consumption coupled to δ^13^C–CH_4_ enrichment (Fig. 1). There was considerable variation in methane oxidation rates between sampled trees of the second experiment, with methane uptake ranging from 3.0 to 81.2 µmol m^−2^ bark d^−1^ (Supplementary Table 1) or 16 to 882 µg per kg of bark d^−1^. No methane consumption or fractionation occurred within blank controls and sterilised (microwaved) bark treatments (Fig. 1). The average fractionation factor (α) observed between the bark samples was similar across the three sampled sites (MF1: 1.040 ± 0.013; FF1: 1.031 ± 0.005; FF2: 1.033 ± 0.017; Fig. 1 and Supplementary Table 1). Fractionation factors were generally higher than reported literature values for MOB, including those reported for upland temperate forested soils (α = 1.018–1.022)^39,40^ and tropical forested soils (1.012–1.023)^41^, but were within range of both subtropical wetlands (α = 1.003–1.032)^24,42^ and rice paddies (α = 1.013–1.033)^43,44^ (Supplementary Table 2). Our lab-based fractionation factor α values may be higher due to methane inoculation concentrations differing to natural field conditions^45^ or may reflect the relatively high community abundance of bark-dwelling MOB found in paired samples (see microbial data in Fig. 2).

### Methane oxidation is strongly correlated with MOB abundance in tree bark

Molecular analysis was used to determine the abundance (quantitative PCR) and composition (amplicon sequencing) of the total bacterial communities (*via* universal 16 S rRNA gene) and MOB communities (*via pmoA* gene encoding a particulate methane monooxygenase subunit) within lower stem bark samples (*n* = 14). The marker gene for aerobic methanotrophy (*pmoA*) was detected in relatively high abundance in every sample (av. 2 × 10^9^ copies per gram of dry sample material; range of 4 × 10^7^ to 5 × 10^9^; Fig. 2a), with values comparable to wetland sediments^46^. The relative abundance of MOB was remarkably high within the bark microbial communities (5.4 to 24.7% based on qPCR, Fig. 2c; 3.2 to 12.8% based on amplicon sequencing, Fig. 2d). This is in stark contrast to the reported low MOB abundance in the heartwood and sapwood of other tree species (<0.1%, *Populus deltoides*)^30^. Compositional profiling revealed that the bark samples hosted unique microbial communities that were distinct from those in adjacent sediments and waters (Fig. 2b and Supplementary Fig. 2; *p* < 0.001) and likely adapted to the acidic pH observed in the bark^47^. Over half of the total bacterial community comprised five genera, *Mycobacterium*, *Acidocella*, *Occallatibacter*, *Conexibacter* and the MOB genus *Methylomonas* (Fig. 2f and Supplementary Fig. 2). Consistently, *Methylomonas* accounted for the majority of the methanotrophic community based on analysis of the total bacterial community (Fig. 2e) and MOB community (Supplementary Fig. 3). Other acidophilic members of the genus are known to be associated with *Sphagnum* mosses and have been shown to significantly mitigate methane emissions from wetlands^38,47^, suggesting *Methylomonas* are well-adapted to vegetation-associated lifestyles. Several trees also hosted a large proportion of novel lineages of Methylacidiphilaceae, a family of acidophilic methanotrophs from the phylum Verrucomicrobia^48–50^ (Fig. 2e), potentially expanding the tree MOB niche to two phyla. Remarkably, MOB abundance determined by qPCR and 16 S rRNA amplicon sequencing strongly predicted methane oxidation rates with paired bark samples (T1–T7) from FF2 (*R*^2^ = 0.76 and 0.74, respectively; Fig. 2c, d). Thus, tree-bark methane oxidation rates are well-explained by the high yet variable abundance of bark-associated methanotrophs dominated by the genus *Methylomonas* (Fig. 2e).

### Field-based MOB inhibition confirms methane sink activity within bark

**Fig. 3:**
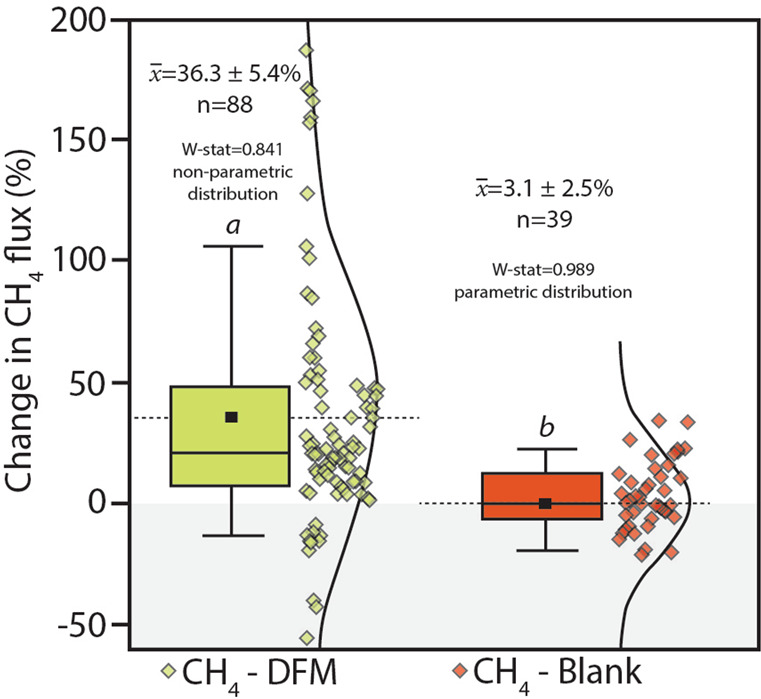
Summary of in situ methanotroph (MOB) inhibitor tests conducted using difluoromethane (DFM) on *M. quinquenervia* bark revealing the mostly positive % increase in methane fluxes ~1 h after the addition of DFM and non-parametric distribution (Shapiro–Wilk, *W*-stat = 0.841). The blank replicates (i.e. repeated chamber measurements after ~1 h, but no DFM addition) showed no change in mean methane fluxes (3.1 ± 2.5%) and normal distribution (Shapiro–Wilk, *W*-stat = 0.989). There were significant differences between treatments *a* and *b* (ANOVA on-ranks, *p* < 0.001). Note: The box represents the 25–75 percentile, error bars 1–99 percentile, the solid horizontal line is the median, dashed line and small square = mean (*x̅*) and the curved line and scatter plots show the data distribution.

To both confirm and quantify the MOB activity moderating tree stem methane emissions in situ, we utilised DFM inhibition experiments on *M. quinquenervia* lower stems (*n* = 88). The use of specific inhibitors of methanotrophy enable estimation of methane oxidation rates by MOB under both lab- and field-based conditions^35,36,51,52^. Low concentrations of difluoromethane (CH_2_F_2_; DFM) temporarily and effectively inhibit methanotrophy by competing with methane as a substrate for methane monooxygenase^36,53^ (the major enzyme catalysing aerobic methane oxidation) without affecting methanogenesis^35^. To achieve this, replicate baseline tree stem methane fluxes were measured before (Supplementary Fig. 1a) and then ~1 h after the addition of DFM (Supplementary Fig. 1b) into tree flux chambers^34^ (see Supplementary Methods for more information). A net positive change in methane fluxes was observed in nearly all chambers after the addition of DFM (average increase of 36.3 ± 5.4%), indicating MOB were present, active and effectively inhibited (Fig. 3). The changes in blank (control) repeated chamber measurements (*n* = 39), without the addition of DFM over a similar incubation period, were normally distributed around zero (mean of 3.1 ± 2.5%) and significantly different to the MOB inhibited DFM measurements (*p* < 0.001, Fig. 3). The change in methane flux rates ranged between −55 to 187% and −36 to 35% for the DFM and control experiments respectively. In some cases, outlier values (both positive and negative) were at locations of lowest methane fluxes, which are most sensitive to subtle variability. Although environmental conditions were relatively stable during all DFM experiments (Supplementary Table 3) and each measurement completed within ~1 h, we cannot rule out that some temporal variability of both methane oxidation and production may occur. Overall, these results provide a first order estimate of in situ bark dwelling MOB activity mitigating ~36% of the methane emissions from *M. quinquenervia* tree stems.

### Future frontiers

Overall, this study provides conclusive evidence of active methane oxidation by a unique MOB community within the bark of a widely distributed lowland tree species. This important discovery adds to our evolving understanding of tree mediated methane fluxes. If MOB are a ubiquitous feature within the bark of methane emitting trees, our conceptual understanding of the global methane cycle may need revision. However, as the *M. quinquenervia* bark structure represents an ideal methane transport medium^12^ and a potentially unique habitat for MOB communities, further work on a variety of tree species featuring more common dense and woody bark substrates is required to determine the generalisability of these findings. Future work in this frontier research area should also focus on (i) constraining MOB importance and magnitude of MOB in mitigating methane emissions from trees, (ii) investigating MOB ecophysiology through metagenomic, imaging and cultivation studies and (iii) determining MOB spatial and geographical distribution from tree scale to global scale, respectively.

## Methods

### In situ tree stem methane flux rates and bark preparation

*M. quinquenervia* lower stem fluxes from forests in subtropical north-eastern New South Wales (NSW), Australia were determined using a small chamber directly attached to the tree and connected with a portable cavity ring-down spectrometer (CRDS, G4301-GasScouter, Picarro) using the ‘Small Nimble In situ Fine-scale Flux’ (S.N.I.F.F.) method^34^. Briefly, a small 50 mm wide PVC chamber was attached to tree stems using white potting clay to create an airtight seal with the stem surface. The SNIFF stem chamber was then connected to the CRDS inlet using a 2 m length of gas tubing (Bev-A-line IV^TM^) via a drying agent (Drierite), with the gas stream then returned from the CRDS outlet to the SNIFF chamber, to close the loop. After 2 min incubations, the CH_4_ flux rate (ppm sec^−1^) was converted to an areal CH_4_ flux (mmol m^−2^ d^−1^) using the flux equation:

1 $$F = \left[ {s\left( {V/RT_{air}A} \right)} \right]t$$

where *s* is the regression slope (ppm s^−1^), *V* is the closed loop volume (m^3^), *R* is the universal gas constant (8.205 × 10^−5^ m^3^ atm K^−1^ mol^−1^) and *T*_air_ is the air temperature inside the chamber (K), *A* is the measured surface area of the clay ring after each measurement (m^2^) and *t* is the conversion from seconds to days.

During the first stable isotope MOB laboratory experiment, two bark samples were collected from lower stem heights from opposite sides of four *M. quinquenervia* trees with high methane fluxes, spanning two sites with differing hydrological characteristics (Supplementary Table 1). One site featured moist sediments (MF1) around the tree base (T8–T11, *n* = 4) whereas the other (FF1) was completely inundated with freshwater ~50 cm up the tree stem (T12–T16, *n* = 5). Samples T15 and T16 were paired samples collected from the same tree on the same side (Supplementary Table 1). The bark swatches were cut using a sterile razor to include all layers from the outer bark to the heartwood surface. The average depth of bark sample collected was 1.3 ± 0.1 cm and ranged from 0.8 to 1.9 cm. Within 1 h of bark sample collection, each sample was weighed (with samples ranging from 81 to 147 g; Supplementary Table 1) and then volumetrically measured with a ruler (cm^3^). The bark samples were then cut into sufficiently narrow strips (~1 cm) to fit through the bottle-neck of sterile (autoclaved) 550 mL crimp top glass bottles. Care was taken to ensure minimal disturbance to the planar bark layers to preserve as much of the natural bark microstructure as possible. Each bottle was then capped, wrapped in aluminium foil and injected with 101 ppm CH_4_ in air gas standard (complete composition = 101 ppm CH_4_, 21% O_2_, balance N_2_; CoreGas). This was achieved by flushing each bottle for 6 min using a two-syringe system, featuring a long inlet syringe reaching near the bottom of the bottle and a short venting syringe evacuating the headspace closer to the top of each bottle. Four bark-free empty bottles (blanks) were used as controls, and were also wrapped, crimped and flushed using the same 101 ppm CH_4_ standard and methods.

A repeat experiment (FF2), focused solely on an inundated forest site, utilised seven trees spanning a range in CH_4_ flux rates (1.1 to 393 mmol m^−2^ d^−1^) (Supplementary Table 1). Larger bark samples (~13 × 25 cm), collected from the lower stem of trees (Supplementary Table 1) in standing water that was on average 54.0 ± 12.9 cm deep, were extracted using sterile methods and then cut into thirds. For the microbial analysis, one third of each bark sample was field-wrapped in sterile foil pockets (pre-baked at 180 °C for 6 h) immediately after extraction, and then placed on ice (*n* = 7). Ancillary sediment and surface water samples were also collected using sterile methods. The composite homogenised sediment samples (*n* = 2) were extracted from various depths of two freshly dug holes (to 20 cm depth), located at the edge of the wetland and within <9.0 m proximity of all sampled trees. Composite homogenised water samples (*n* = 2) were collected directly from 10 cm below the water surface using sterile syringes from several locations nearby the inundated trees at undisturbed sites. All samples were refrigerated within 2 h of collection at 4 °C. They were later transported with dry ice to Monash University (Greening Lab) for the microbial analysis. One third of each bark sample was prepared as per first experiment methods (*n* = 7) and were placed into sealed crimp top sterile bottles. The final third of bark sample from each tree were placed in sterile crimp top bottles, but microbial communities were neutralised by microwaving (1600W – LG model MS3882XRSK) for 2 min, four times over, before sealing (*n* = 7). All paired samples (i.e. raw bark and microwaved control) were then inoculated with 101 ppm methane as per the syringe method above.

### Isotope time series inoculation experiment

The headspace concentration of CH_4_ and δ^13^C–CH_4_ of the inoculated bark bottles, the neutralised bark bottles and blanks were sampled using a CRDS with a sensitivity of 5 ppb + 0.05% of reading for  ^12^C and 1 ppb + 0.05% of reading for ^13^C (Picarro, G2201-i). At 3 to 24 hourly intervals (increasing with experiment duration), a 60 mL gas sample of 101 ppm CH_4_ was injected into the bottle septum using a long syringe needle, whilst simultaneously mixing and removing 60 mL of gas sample via a second and short syringe needle. To ensure adequate headspace mixing occurred, headspace mixing was repeated at least eight times before extracting each sample (i.e. the volume of gas mixed was greater than the headspace volume within each bottle). The extracted gas sample was then analysed directly from the syringe into the CRDS. The sample concentration of CH_4_ (ppm), δ^13^C–CH_4_ (‰) and the associated ±SD were recorded for each bottle treatment at each time interval. The 60 mL mixing additions of CH_4_ and δ^13^C–CH_4_ (‰) to each bottle headspace were later accounted for via mass balance, to calculate the shift in CH_4_ and δ^13^C–CH_4_ (‰) over time. The decrease in CH_4_ over the first 24 h was converted to uptake, as a proportion of the original surface area of each bark treatment within each bottle. The fractionation factor (α) was defined as the ratio of the oxidation rate coefficients of ^12^CH_4_ over ^13^CH_4_, and calculated using established methods^54^.

### Genomic DNA extraction

High-quality and amplifiable genomic DNA were extracted from all bark (*n* = 14), sediment (*n* = 3) and water (*n* = 6) samples. For each individual bark sample, 0.13 to 0.18 g (wet weight) of material was frozen in liquid nitrogen and immediately homogenised using a sterile pestle and mortar until a fine powder was obtained. Genomic DNA was extracted from the homogenised samples using the Synergy 2.0 Plant DNA Extraction Kit (OPS Diagnostics LLC, US), according to the manufacturer’s instructions. Genomic DNA from the sediment samples (0.25 g wet weight sample) and water samples (50 mL sample filtered on to sterile filter papers) were extracted using the DNeasy PowerSoil Kit (Qiagen, US), according to the manufacturer’s instructions. The purity and yield of the DNA extracts were verified by spectrophotometry (NanoDrop ND-1000 spectrophotometer, Nanodrop Technologies Inc., US) and quantified by fluorometry (Qubit Fluorometer, Thermo Fisher Scientific). For the DNA extraction from each type of sample, PCR-grade water was extracted as a negative control.

### Quantitative PCR

Quantitative PCR assays were performed on a QuantStudio 7 Flex Real-Time PCR instrument (Thermo Fisher Scientific) in order to quantify gene copy numbers and estimate the abundance of the total microbial (16 rRNA gene copies) and MOB community (*pmoA* gene copies). Briefly, the *pmoA* gene was amplified using the previously described degenerate primers A189f 5′-GGNGACTGGGACTTCTGG-3′ and mb661 5′-CCGGMGCAACGTCYTTACC-3′^55,56^ and cycling conditions^57^. The primer pair was chosen for its coverage of the MOB community from environments with elevated CH_4_ concentrations. The V4 hypervariable region of the 16 S rRNA gene was amplified using the universal Earth Microbiome Project primer pairs 515FB 5′-GTGYCAGCMGCCGCGGTAA-3′ and 806RB 5′-GGACTACNVGGGTWTCTAAT-3′^58^, as per previously described cycling conditions^59^. The employed reaction conditions and thermal profiles of the qPCR assays have been previously described^37^. Amplification from different dilutions (from undiluted to 1:100 dilution in PCR-grade water) of DNA extracts was tested, and the dilution resulting in the highest yield and quality of PCR product was used for the qPCR assays. For each assay (96-well plate), duplicate serial dilutions of quantified 16 S rRNA gene (from *Escherichia coli*) or *pmoA* gene amplicons, (from *Methylosinus trichosporium* strain OB3b) were used to generate standard calibration curves. Each sample was analysed in triplicate; amplification efficiencies (>70%) were calculated from the slopes of the calibration curves (*R*^2^ values >0.97). No significant amplification of the blank extractions was observed in any qPCR assays.

### Amplicon sequencing

Amplicon sequencing of the universal 16 S rRNA gene was used to infer the community composition of the total bacterial and archaeal community within each sample. Amplicon sequencing of the *pmoA* gene, encoding the particulate methane monooxygenase A subunit, was also performed to gain a higher-resolution insight into the composition of the MOB community. The same primer pairs used for the quantitative PCR assays (reported above) have been employed in the amplicon sequencing of the 16 S rRNA and pmoA genes. Genomic DNA extracts of 14 bark, two composite sediment and two composite water samples (pooled samples), as well as the blank extraction, were subject to Illumina paired-end sequencing at the Australian Centre for Ecogenomics, University of Queensland. The resultant raw sequences from the 16 S rRNA gene amplicon sequencing were subject to quality filtering, merging, primer trimming, denoising and singleton removal using the QIIME 2 platform^60^. Taxonomic affiliation of the identified amplicon sequence variants (16S-ASVs) was assigned according to the GTDB taxonomy^61^, release 05-RS95. For each sample, 16S-ASVs classified as ‘unassigned' (av. 5.2 %), ‘Eukaryota' (av. 0.04%), ‘Chloroplast' (av. 1.7%) and ‘Mitochondria' (av. 0.3%) were excluded as being potentially derived from plant material. The final dataset accounted for 2727 16S-ASVs, with an average sequence count number per sample of 9184 (range 3657 in sample T6.2 to 14524 in sample S2). The 16S-ASVs assigned to known methanotrophic families and genera were subset to infer MOB community structure and to estimate the proportion of the MOB community within the total microbial community via the 16S-ASV dataset. Note that this analysis cannot detect uncultured MOB with unknown 16 S rRNA gene sequences. Data processing of the *pmoA* gene amplicon sequences followed our previously published pipeline^37,62^, with minor modifications. All processing steps were performed in the QIIME 2 platform and, instead of assigning the raw sequences to operational taxonomic units, raw sequences were denoised using the DADA2 pipeline^63^, yielding 280 high-quality *pmoA* amplicon sequence variants (*pmoA*-ASVs). Taxonomic affiliation of the *pmoA*-ASVs was assigned by similarity with *pmoA* sequences of a curated database^64^. The average sequence count number per sample was 8556; range 5026 in sample T7.1 to 14,729 in sample T5.1. Sample T6.2, with a sequence count number of 1503, was excluded from further analyses. Note that this analysis cannot detect highly divergent MOB *pmoA* sequences, such as those from Verrucomicrobiota and *Candidatus* Methylomirabilota.

### Microbial diversity analyses

To assess total and MOB community structure based on both 16S-ASV and *pmoA*-ASV dataset, read count normalisation and alpha and beta diversity calculations were performed with the package phyloseq v1.30^65^ from the open source software Bioconductor. Chao1, Shannon and Inverse Simpson indices were computed to assess the alpha diversity of total and MOB communities, whereas beta diversity was measured using the Bray–Curtis distance matrix^66^ and visualised using non-parametric multidimensional scaling ordinations (nMDS). To determine whether the observed between-group distances were statistically significant, we performed permutational multivariate analysis of variance (PERMANOVA) with the software PRIMER-E v7 (PRIMER-E Ltd., Plymouth, United Kingdom). For bark samples, correlations between *pmoA* and 16 S rRNA gene abundance, qPCR- and 16S-ASV-based MOB community proportion, and CH_4_ uptake and in situ tree stem CH_4_ fluxes were tested for significance using linear regression, after appropriate variable transformations (log_10_ for gene abundances, logit for MOB community proportion) and adequate model diagnostic during which we checked for normality, independency between observations and for influential data points (via Leverage and Cook’s distance plots). No data point were excluded. Correlations between qPCR- and 16S-ASV-based MOB community proportion and CH_4_ uptake were highly significant (*p* < 0.008).

### In situ methanotroph inhibitor experiments with DFM

The DFM experiments were conducted during daytime in winter, under dry sunny conditions between the 29 June–13 August 2020. Ambient air temperature amplitude varied minimally during each sample day and across the entire sampling period, increasing by an average of 2.5 ± 0.9 °C between 9:00–15:00, when conducting the stem methane flux experiments (Supplementary Table 3)^67^. The in situ MOB oxidation rates were estimated by first measuring duplicate lower tree stem fluxes (<50 cm above the water level) using the S.N.I.F.F. method as mentioned above^34^ (*n* = 88 trees). Then the tree stem chamber was flushed with atmospheric air for 30 s or until atmospheric concentration in the chamber was attained, and 120 mL addition of 2% DFM was slowly injected, then sealed within each chamber and left to incubate and infiltrate the bark for ~45–90 min, similar to a sufficient time previously shown to inhibit MOB^35^. The chamber was then again flushed again with atmospheric air and then duplicate methane flux rates were measured. Rapid stem flux measurements were possible using 2 min incubations, due to the small S.N.I.F.F chamber volume, CRDS sampling frequency and the high methane emissions from the flooded forest tree stems (average flux rate linear regression *r*^2^ = 0.985 ± 0.006, *n* = 88 trees). As DFM has been shown to be an effective inhibitor of aerobic methanotrophy^36^, the difference between initial methane fluxes and the subsequent DFM inoculated fluxes were deemed to be the effect of inhibition of MOB^53^. Blank repeated chamber flux measurements with no DFM injections were also performed in situ to ensure no enhancement of methane fluxes occurred, as a result of repeated chamber measurement at the same location (*n* = 39). Closed loop experiments conducted in the laboratory spanning a spectrum of methane concentrations (1.8–400 ppm) revealed no increase in methane concentrations occurred when adding 2% DFM when using a CRDS (Picarro, GasScouter G4301). Occasionally, interference with the H_2_O sensor was observed, but never under field conditions when DFM was left to incubate and diffuse. Shapiro–Wilk normality tests were used to determine whether the percentage change in DFM and blank repeat CH_4_ flux treatments were non-parametric (*p* < 0.05) and had equal variance (*p* < 0.05), using Sigmaplot 13.0. A Kruskal–Wallis one aay analysis of variance on ranks was then used to determine whether there was a significant difference between the treatments and Dunn’s method was then used to isolate the group/s that differed from the others, using pairwise multiple comparison procedures, where statistically significant differences were *p* < 0.001.

### Reporting Summary

Further information on research design is available in the Nature Research Reporting Summary linked to this article.

## Supplementary information

Supplementary Information

Reporting Summary

## Data Availability

All sequence data generated and analysed in this study have been deposited at the Sequence Read Archive database and are publicly available under BioProject accession number PRJNA669491. Taxonomic affiliation of the identified amplicon sequence variants (ASVs) was assigned using the GTDB taxonomy database release 05-RS95 (for 16 S rRNA), and the curated database can be found at 10.5880/GFZ.5.3.2016.001 (for *pmoA* ASVs). The lab based MOB inoculation experiment and field based DFM MOB inhibition experiment data that support the findings of this study are available at https://data.mendeley.com/datasets/tw7g2gczwb/1.

## Code availability

The R script used to calculate methane gas flux rates are available in https://data.mendeley.com/datasets/x2sf762mhj/1. The diversity analysis and community profiling were determined using Phyloseq package R v1.30.1 (publicly available as part of the open-source package) and the statistical analysis of diversity sequencing data were determined using PRIMER-e version 7 software (commercially available).
