# Supplementary material for: Bark-dwelling methanotrophic bacteria decrease methane emissions from trees
Source: Nat Commun. 2021 Apr 9;12:2127. doi: 10.1038/s41467-021-22333-7 (PMC8035153; doi:10.1038/s41467-021-22333-7)
Supplement: Supplementary file 1 — Supplementary Information [file 41467_2021_22333_MOESM1_ESM.pdf]

**Supplementary material for**

# Bark-dwelling methanotrophic bacteria decrease methane emissions from trees

Luke C. Jeffrey[1], Damien T. Maher[1,2], Eleonora Chiri[3], Pok Man Leung[3], Philipp A. Nauer[4,5], Stefan K. Arndt[5], Douglas R. Tait[1], Chris Greening[3] & Scott G. Johnston[1]

[1] Southern Cross Geoscience, Southern Cross University, Lismore, NSW, 2480, Australia.

[2] Environment, Science and Engineering, Southern Cross University, Lismore NSW, 2480, Australia.

[3] Department of Microbiology, Biomedicine Discovery Institute, Monash University, Clayton, VIC, 3800, Australia

[4] School of Chemistry, Monash University, Clayton, VIC, 3800, Australia

[5] School of Ecosystem and Forest Sciences, University of Melbourne, Richmond, VIC, 3121, Australia

Key points of the manuscript:

- The bark of a common wetland tree species contains a unique microbial community containing up to 25 % methane-oxidising bacteria (MOB)
- Bark-dwelling MOB can decrease tree methane emissions by ~ 36 %
- MOB abundance in bark strongly predicted methane oxidation rates
- Tree bark MOB represent a previously unrecognised methane sink

## Abstract

Tree stems are an important and unconstrained source of methane, yet it is uncertain if there are internal microbial controls (i.e. methanotrophy) within tree bark that may reduce methane emissions. Using multiple lines of evidence, here we demonstrate that unique microbial communities dominated by methane-oxidising bacteria (MOB) dwell within bark of *Melaleuca quinquenervia*, a common, invasive and globally distributed lowland species. In laboratory incubations, methane-inoculated *M. quinquenervia* bark mediated methane consumption (up to 96.3 µmol $m^{-2}$ bark $d^{-1}$) and there was a distinct isotopic $\delta^{13}C\text{-}CH_4$ enrichment characteristic of MOB. Molecular analysis indicates unique microbial communities reside within the bark, with methane-oxidising bacteria primarily from the genus *Methylomonas* comprising up to 25 % of the total microbial community. Methanotroph abundance was linearly correlated to methane uptake rates ($R^2 = 0.76$, $p = 0.006$). Finally, field-based methane oxidation inhibition experiments demonstrate that bark-dwelling MOB reduce methane emissions by 36 ± 5 %. These multiple complementary lines of evidence indicate that bark-dwelling MOB represent a potentially significant methane sink, and an important frontier for further research.

## Supplementary Tables.

**Supplementary Table 1.** Summary of sampled trees, *in situ* parameters and bark samples of the two time series MOB incubation experiments indicating the methane uptake (µmol m$^{-2}$ bark d$^{-1}$) and fractionation factor (α) measured under laboratory conditions.

| Site | Tree ID | CBH (cm) | Bark sample height (cm> sediment) | Water depth to tree base (cm) | Bark wet weight (g) | Bark water content (%) | Bark surface area (cm$^2$) | *In situ* tree stem $CH_4$ flux (mmol m$^{-2}$ d$^{-1}$) | $CH_4$ uptake first 24 h (µmol m$^{-2}$ bark d$^{-1}$) | Fractionation factor (α) |
|---|---|---|---|---|---|---|---|---|---|---|
| Moist Forest (MF) | T8* | 77 | 30-45 | 5 | 102.0 | - | 90.5 | - | -26.1 | 1.043 |
| | T9* | 77 | 30-45 | 5 | 81.3 | - | 80.3 | | -19.9 | 1.029 |
| | T10‡ | 70 | 45-60 | 0 | 95.4 | - | 78.0 | - | -20.9 | 1.058 |
| | T11‡ | 70 | 45-60 | 0 | 92.1 | - | 71.2 | | -39.8 | 1.030 |
| Flooded Forest 1 (FF1) | T12§ | 103 | 74-90 | 61 | 109.0 | - | 91.0 | - | -34.5 | 1.027 |
| | T13§ | 103 | 74-90 | 61 | 113.2 | - | 114.0 | | -32.8 | 1.031 |
| | T14† | 112 | 58-70 | 42 | 106.1 | - | 74.9 | - | -96.3 | 1.026 |
| | T15† | 112 | 58-70 | 42 | 147.6 | - | 68.1 | | -20.8 | 1.038 |
| | T16† | 112 | 58-70 | 42 | 126.3 | - | 48.0 | | -63.9 | 1.033 |
| Flooded Forest 2 (FF2) | T1 | 80 | 48-63 | 40 | 108.0 | 79.9 | 71.9 | 138.0 | -3.0 | 1.006 |
| | T2 | 75 | 56-68 | 46 | 112.4 | 84.6 | 128.7 | 1.1 | -4.8 | 1.000 |
| | T3 | 57 | 70-82 | 61 | 110.0 | 80.6 | 106.8 | 256.5 | -45.0 | 1.035 |
| | T4 | 55 | 74-90 | 65 | 75.0 | 79.5 | 104.1 | 18.6 | -81.2 | 1.031 |
| | T5 | 72 | 81-93 | 64 | 100.1 | 79.0 | 123.2 | 380.4 | -63.0 | 1.031 |
| | T6 | 56 | 43-56 | 36 | 98.5 | 77.3 | 109.7 | 239.4 | -13.3 | 1.046 |
| | T7 | 73 | 74-93 | 66 | 116.1 | 80.6 | 159.0 | 393.1 | -78.1 | 1.035 |
| Average | | 82 | | 40 | 105.8 | 79.4 | 95.0 | 203.9 | -40.2 | 1.031 |
| ±SD | | 20 | | 24 | 17.0 | 1.34 | 28.0 | 158.5 | 28.5 | 0.014 |
| Min | | 55 | | 0 | 75.0 | 77.3 | 48.0 | 1.1 | -3.0 | 1.000 |
| Max | | 112 | | 66 | 147.6 | 84.6 | 159.0 | 393.1 | -96.3 | 1.058 |

Note: *‡§†symbols denote bark samples taken from the same tree on opposing sides

**Supplementary Table 2.** Range of literature reported methane oxidation fractionation factors (α) from a variety different ecosystems.

| α Minimum | α Maximum | α Average | Study design | Authors[citation] |
|---|---|---|---|---|
| 1.002 | 1.014 | | Sediment & water column review | Whiticar & Faber (1986)[1] |
| - | - | 1.011 | Methods comparison | Silverman & Oyama (1968)[2] |
| - | - | 1.030 | - | Zyakun *et al.* (1979)[3] |
| 1.005 | 1.031 | - | Groundwater methane oxidation | Barker & Fritz (1981)[4] |
| 1.013 | 1.025 | - | - | Coleman *et al.* (1981)[5] |
| 1.007 | 1.027 | - | Natural ecosystems | Zyakun *et al.* (1983 [6] |
| - | - | 1.037 | Polyhumic lake | Kankaala *et al.* (2007)[7] |
| 1.017 | 1.028 | 1.023 | Temperate upland forested soils | Tyler *et al.* (1994)[8] |
| 1.003 | 1.021 | - | Swamp forests | Happell *et al.* (1994)[9] |
| 1.027 | 1.032 | 1.030 | Freshwater wetland water column | Jeffrey *et al.* (2019)[10] |
| 1.025 | 1.049 | - | Landfill cover soils | Chanton & Liptay (2000)[11] |
| 1.002 | 1.030 | - | Lab based cultures | Templeton *et al.* (2006)[12] |
| - | - | 1.021 | Rice paddy soils | Zhang et al. (2016)[14] |
| - | - | 1.013 | Rice paddy Roots | Zhang *et al.* (2016)[14] |
| - | - | 1.025 | Rice paddies | Zhang et al. (2013)[13] |
| - | - | 1.033 | Rice paddies with straw | Zhang *et al.* (2013)[13] |
| 1.012 | 1.039 | - | Marine habitats | Holler *et al.* (2009)[16] |
| - | - | 1.017 | Grassland soils | Snover & Quay (2000)[15] |
| - | - | 1.018 | Temperate forested soils | Snover & Quay (2000)[15] |
| 1.021 | 1.022 | - | Mineral soils | Maxfield *et al.* (2008)[18] |
| 1.012 | 1.023 | - | Humid tropical forested soils | Teh *et al.* (2005)[17] |
| - | - | 1.008 | Landfill soils | Bergamaschi *et al.* (1998)[19] |
| 1.023 | 1.026 | - | Boreal forested soils | Reeburgh *et al.* (1997)[20] |
| 1.016 | 1.027 | - | Tundra soils | King *et al.* (1989)[21] |
| 1.029 | 1.058 | 1.040 | *M. quinquenervia* bark | MF1 - This study |
| 1.026 | 1.038 | 1.031 | *M. quinquenervia* bark | FF1 - This study |
| 1.000 | 1.046 | 1.026 | *M. quinquenervia* bark | FF2 - This study |

**Supplementary Table 3.** Ancillary environmental parameters during field sampling.

| Experiment | Sampling date | Overnight Minimum Temp (°C) | Temperature range during sampling (°C) | Δ Air temperature during sampling (°C) | Rainfall 48 h prior to sampling (mm) | 9am wind speeds (km/h) |
|---|---|---|---|---|---|---|
| MF1 & FF1 Experiment | 4/05/2020 | 14.8 | 19.5 - 21.6 | 2.1 | 0 | 20 |
| FF2 Experiment | 20/05/2020 | 15.1 | 17.9 - 19.1 | 1.2 | 16.2 | 6 |
| DFM - day 1 | 29/06/2020 | 11.3 | 16.7 - 19.1 | 2.4 | 0 | 15 |
| DFM - day 2 | 30/06/2020 | 11.6 | 16.5 - 20.1 | 3.6 | 1.4 | 15 |
| DFM - day 3 | 6/07/2020 | 7.0 | 16.8 - 20.2 | 3.4 | 0.2 | 19 |
| DFM - day 4 | 13/08/2020 | 14.5 | 20.8 - 23.0 | 2.2 | 0 | 24 |
| Min | | 7.0 | | 1.2 | 0 | 6 |
| Max | | 15.1 | | 3.6 | 16.2 | 24 |
| Average | | 12.4 | | 2.5 | 3.0 | 16.5 |
| SD | | 3.1 | | 0.9 | 6.5 | 6.2 |

## Supplementary Figures

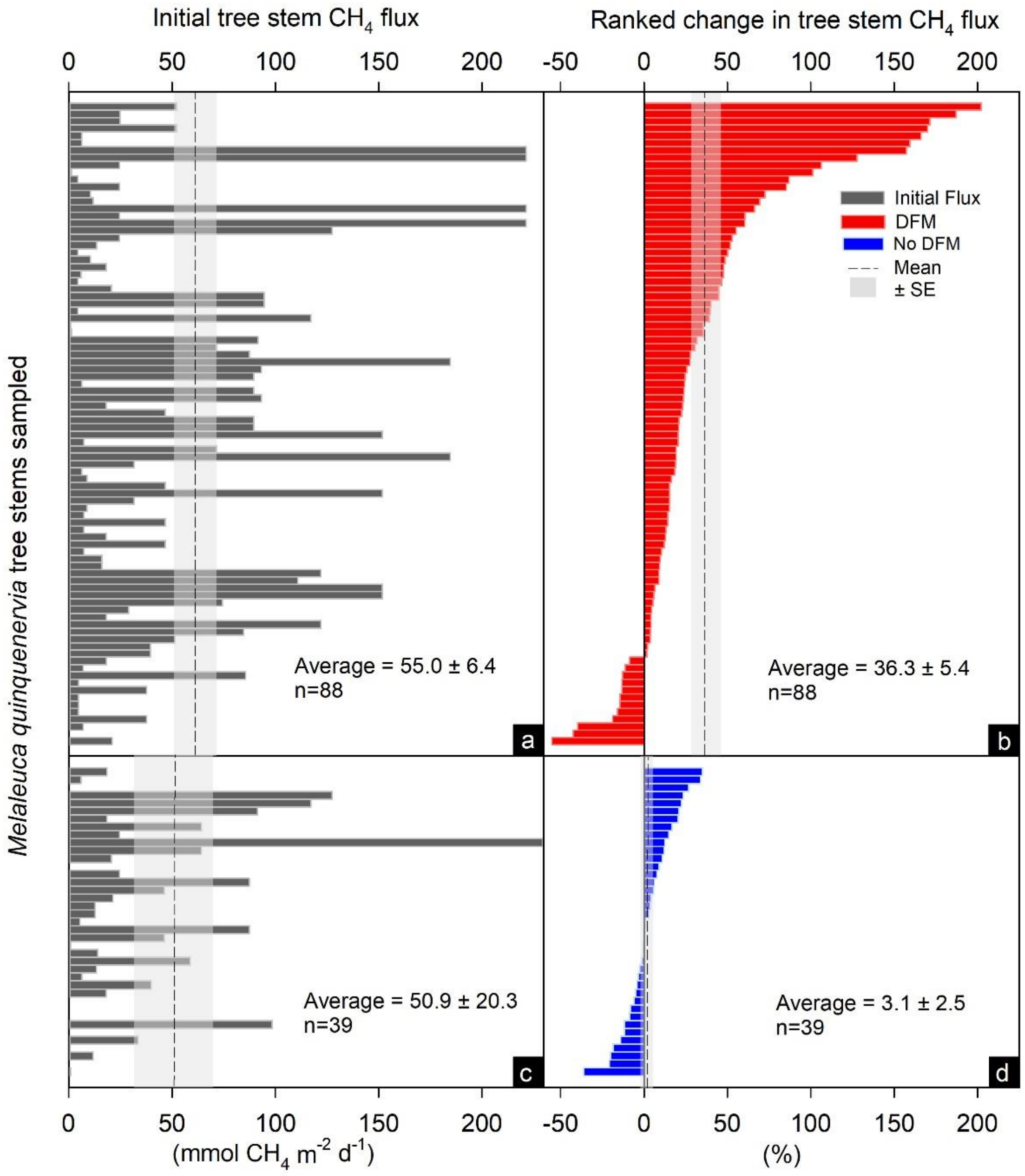


**Supplementary Figure 1.** Results of DFM experiments showing: (a) initial $CH_4$ flux rates from *M. quinquenervia* stem; (b) % change in $CH_4$ emissions ~1 hour after the addition of DFM to the stem chamber; (c) initial $CH_4$ flux rates from *M. quinquenervia* stem for blank treatments; and (d) % change in $CH_4$ emissions ~1 hour blank chamber measurements. Paired data ranked according to % change in $CH_4$ emissions.

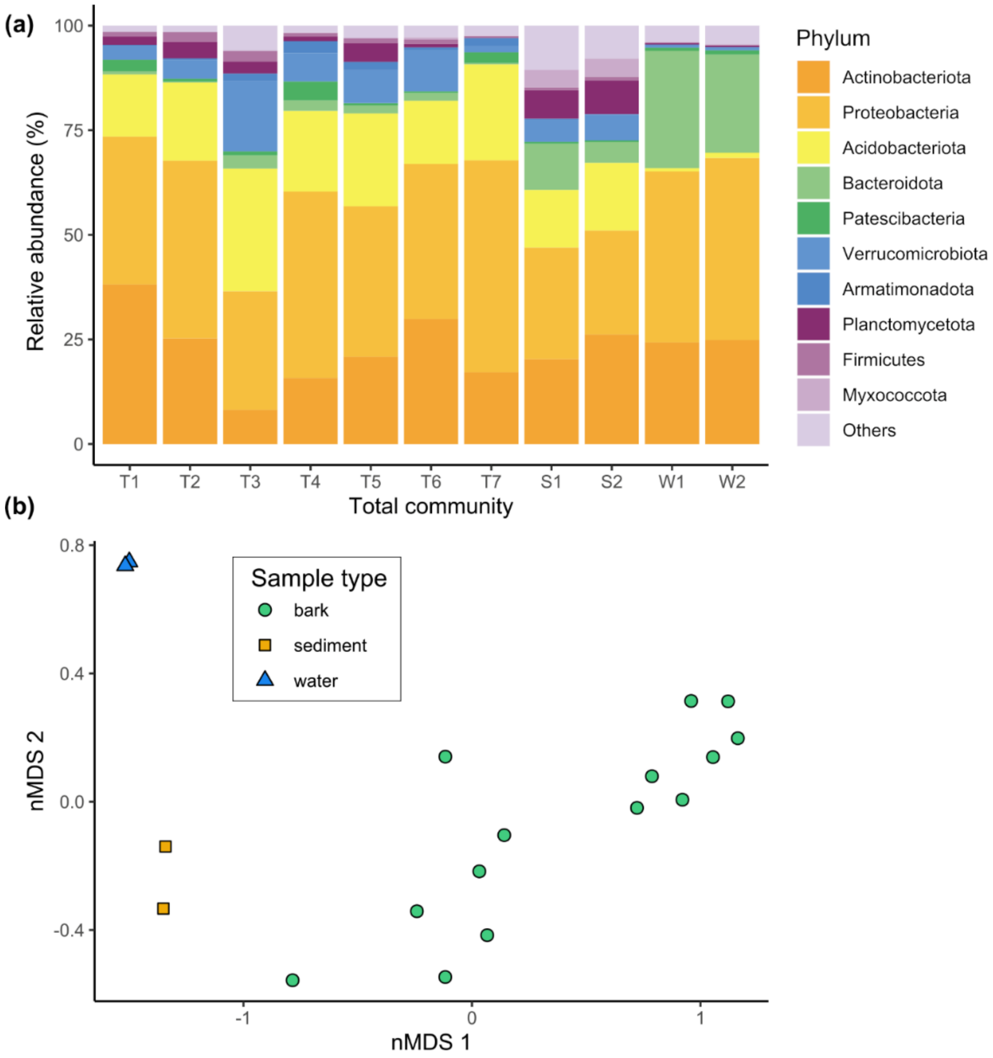


**Supplementary Figure 2. (a)** Relative abundance of 16S rRNA gene amplicon sequences resolved at the taxonomic level of phylum. **(b)** Non-metric multidimensional scaling (nMDS) ordination of the MOB community structure (beta diversity) measured by Bray-Curtis distance matrix of the 16S rRNA gene amplicon sequences.

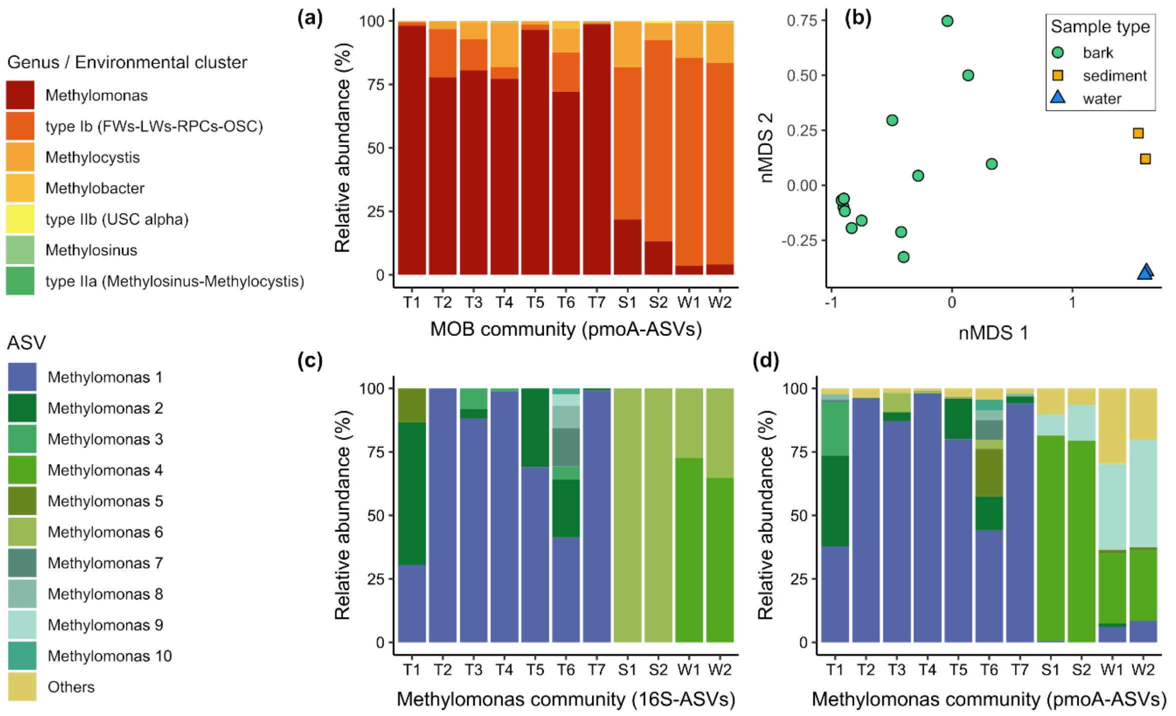


**Supplementary Figure 3. (a)** Relative abundance of methanotrophic (MOB) community genera and uncultivated environmental clusters (fresh water “FWs”, lake water “LWs”, rice field soil “RPCs”, organic soil “OSC”, and upland soil alpha “USC alpha” clusters) detected from the analysis of the *pmoA* gene amplicon sequences. **(b)** Non-metric multidimensional scaling (nMDS) ordination of the MOB community structure (beta diversity) measured by Bray-Curtis distance matrix of the *pmoA* gene amplicon sequences. **(c), (d)** Relative abundance of the *Methylomonas* amplicon sequence variants identified from the analysis of the 16S rRNA gene (c) and *pmoA* (d) amplicon sequences

**Citations**


1 Whiticar, M. J. & Faber, E. Methane oxidation in sediment and water column environments—isotope evidence. *Organic Geochemistry* **10**, 759-768 (1986).

2 Silverman, M. P. & Oyama, V. I. Automatic apparatus for sampling and preparing gases for mass spectral analysis in studies of carbon isotope fractionation during methane metabolism. *Analytical chemistry* **40**, 1833-1837 (1968).

3 Zyakun, A., Bondar, V. & Namsaraev, B. Fractionation of stable carbon isotopes of methane in process of microbiological oxidation. *Geokhimiya;(USSR)* **2** (1979).

4 Barker, J. F. & Fritz, P. Carbon isotope fractionation during microbial methane oxidation. *Nature* **293**, 289-291 (1981).

5 Coleman, D. D., Risatti, J. B. & Schoell, M. Fractionation of carbon and hydrogen isotopes by methane-oxidizing bacteria. *Geochimica et Cosmochimica Acta* **45**, 1033-1037 (1981).

6 Zyakun, A., Bondar, V., Namsarayev, B. & MESTEROV, A. Use of carbon isotope analysis in determining the rates of microbiological oxidation of methane in natural ecosystems. *Geochemistry international* **20**, 67-72 (1983).

7 Kankaala, P., Taipale, S., Nykänen, H. & Jones, R. I. Oxidation, efflux, and isotopic fractionation of methane during autumnal turnover in a polyhumic, boreal lake. *Journal of Geophysical Research: Biogeosciences* **112** (2007).

8 Tyler, S. C., Crill, P. M. & Brailsford, G. W. 13C12C Fractionation of methane during oxidation in a temperate forested soil. *Geochimica et Cosmochimica Acta* **58**, 1625-1633 (1994).

9 Happell, J. D., Chanton, J. P. & Showers, W. S. The influence of methane oxidation on the stable isotopic composition of methane emitted from Florida swamp forests. *Geochimica et Cosmochimica Acta* **58**, 4377-4388 (1994).

10 Jeffrey, L. C. *et al.* Wetland methane emissions dominated by plant-mediated fluxes: Contrasting emissions pathways and seasons within a shallow freshwater subtropical wetland. *Limnology and Oceanography*, doi:10.1002/lno.11158 (2019).

11 Chanton, J. & Liptay, K. Seasonal variation in methane oxidation in a landfill cover soil as determined by an in situ stable isotope technique. *Global Biogeochemical Cycles* **14**, 51-60 (2000).

12 Templeton, A. S., Chu, K.-H., Alvarez-Cohen, L. & Conrad, M. E. Variable carbon isotope fractionation expressed by aerobic CH4-oxidizing bacteria. *Geochimica et Cosmochimica Acta* **70**, 1739-1752 (2006).

13 Zhang, G. *et al.* Pathway of CH 4 production, fraction of CH 4 oxidized, and 13 C isotope fractionation in a straw-incorporated rice field. *Biogeosciences* **10**, 3375-3389 (2013).

14 Zhang, G., Yu, H., Fan, X., Ma, J. & Xu, H. Carbon isotope fractionation reveals distinct process of CH 4 emission from different compartments of paddy ecosystem. *Scientific reports* **6**, 1-10 (2016).

15 Snover, A. K. & Quay, P. D. Hydrogen and carbon kinetic isotope effects during soil uptake of atmospheric methane. *Global Biogeochemical Cycles* **14**, 25-39 (2000).

16 Holler, T. *et al.* Substantial 13C/12C and D/H fractionation during anaerobic oxidation of methane by marine consortia enriched in vitro. *Environmental microbiology reports* **1**, 370-376 (2009).

17 Teh, Y. A., Silver, W. L. & Conrad, M. E. Oxygen effects on methane production and oxidation in humid tropical forest soils. *Global Change Biology* **11**, 1283-1297 (2005).

18 Maxfield, P., Evershed, R. & Hornibrook, E. Physical and biological controls on the in situ kinetic isotope effect associated with oxidation of atmospheric CH4 in mineral soils. *Environmental science & technology* **42**, 7824-7830 (2008).

19 Bergamaschi, P. *et al.* Stable isotopic signatures (δ13C, δD) of methane from European landfill sites. *Journal of Geophysical Research: Atmospheres* **103**, 8251-8265 (1998).

20 Reeburgh, W., Hirsch, A., Sansone, F., Popp, B. & Rust, T. Carbon kinetic isotope effect accompanying microbial oxidation of methane in boreal forest soils. *Geochimica et Cosmochimica Acta* **61**, 4761-4767 (1997).

21 King, S. L., Quay, P. D. & Lansdown, J. M. The 13C/12C kinetic isotope effect for soil oxidation of methane at ambient atmospheric concentrations. *Journal of Geophysical Research: Atmospheres* **94**, 18273-18277 (1989).
