# Supplementary material for: Bark-dwelling methanotrophic bacteria decrease methane emissions from trees
Source: Nat Commun. 2021 Apr 9;12:2127. doi: 10.1038/s41467-021-22333-7 (PMC8035153; doi:10.1038/s41467-021-22333-7)
Supplement: Supplementary file 2 — Reporting Summary [file 41467_2021_22333_MOESM2_ESM.pdf]

nature research

Corresponding author(s): Luke Jeffrey

Last updated by author(s): Mar 3, 2021

Reporting Summary

Nature Research wishes to improve the reproducibility of the work that we publish. This form provides structure for consistency and transparency in reporting. For further information on Nature Research policies, see our Editorial Policies and the Editorial Policy Checklist.

Statistics

For all statistical analyses, confirm that the following items are present in the figure legend, table legend, main text, or Methods section.

n/a	Confirmed	
☐	☒	The exact sample size (*n*) for each experimental group/condition, given as a discrete number and unit of measurement
☐	☒	A statement on whether measurements were taken from distinct samples or whether the same sample was measured repeatedly
☐	☒	The statistical test(s) used AND whether they are one- or two-sided<br>*Only common tests should be described solely by name; describe more complex techniques in the Methods section.*
☐	☒	A description of all covariates tested
☐	☒	A description of any assumptions or corrections, such as tests of normality and adjustment for multiple comparisons
☐	☒	A full description of the statistical parameters including central tendency (e.g. means) or other basic estimates (e.g. regression coefficient) AND variation (e.g. standard deviation) or associated estimates of uncertainty (e.g. confidence intervals)
☐	☒	For null hypothesis testing, the test statistic (e.g. F, t, r) with confidence intervals, effect sizes, degrees of freedom and P value noted<br>*Give P values as exact values whenever suitable.*
☒	☐	For Bayesian analysis, information on the choice of priors and Markov chain Monte Carlo settings
☒	☐	For hierarchical and complex designs, identification of the appropriate level for tests and full reporting of outcomes
☒	☐	Estimates of effect sizes (e.g. Cohen's d, Pearson's r), indicating how they were calculated

Our web collection on statistics for biologists contains articles on many of the points above.

Software and code

Policy information about availability of computer code

Data collection	No custom algorithm/software was use to collect data in this study
Data analysis	The R script used to calculate the DFM gas fluxes is available here: https://data.mendeley.com/datasets/x2sf762mhj/1<br>Algorithms and software used for the analysis of the sequencing data:<br>Diversity analysis and community profiling - Phyloseq package R v1.30.1 (publicly available as part of the open-source package)<br>Statistical analysis of diversity - PRIMER-e version 7 software (commercially available)<br>Sigmaplot 13.0 was used for the DFM and incubation experiment figures, plots and statistical tests

For manuscripts utilizing custom algorithms or software that are central to the research but not yet described in published literature, software must be made available to editors and reviewers. We strongly encourage code deposition in a community repository (e.g. GitHub). See the Nature Research guidelines for submitting code & software for further information.

Data

Policy information about availability of data

All manuscripts must include a data availability statement. This statement should provide the following information, where applicable:

- Accession codes, unique identifiers, or web links for publicly available datasets
- A list of figures that have associated raw data
- A description of any restrictions on data availability

All DFM experiment data, incubation experiment data and the microbial genomic sequences will be made available through online repositories released upon publication. All gene sequences for this project have been deposited at the Sequence Read Archive and can be accessed with accession number PPRJNA669491. Sequences will be released upon publication.

Field-specific reporting

Please select the one below that is the best fit for your research. If you are not sure, read the appropriate sections before making your selection.

☐ Life sciences ☐ Behavioural & social sciences ☒ Ecological, evolutionary & environmental sciences

For a reference copy of the document with all sections, see nature.com/documents/nr-reporting-summary-flat.pdf

Ecological, evolutionary & environmental sciences study design

All studies must disclose on these points even when the disclosure is negative.

Study description	Using multiple lines of investigation, this study determined whether bark-dwelling methane oxidising bacteria (MOB) live in wetland lowland trees. Lab based bark methane inoculation experiments (n=9) revealed isotopic fractionation and methane reduction indicative of MOB. Repeated paired experiment (n=7) confirmed this result, with the analysis of the microbial community confirming that bark dwelling MOB (n=14) were highly abundant. Field observations comparing tree stem CH4 fluxes (n=88) before and after the addition of MOB inhibitor (Difluoromethane gas - DFM) showed fluxes increased by 36% after the addition of DFM, confirming that bark-dwelling MOB were present and active in situ.
Research sample	Melaleuca quinquenervia (Broadleaf paper bark tree) are a common lowland forest species capable of emitting high stem fluxes of methane. They represent a globally distributed and invasive species, commonly found and native to subtropical Australia. This species was selected as a likely host of bark-dwelling MOB due to their often waterlogged environments conducive to methane production, their unique bark substrate and high reported stem methane fluxes. We also compared MOB microbial community abundance to the surrounding soil and water column.
Sampling strategy	Testing our hypothesis that bark-dwelling MOB exist, relied upon testing the differences between lab based treatments and through microbial genomic extractions. The 16 bark samples from 11 trees of differing hydrological forest locations was sufficient to determine a difference between the methane consumption in inoculated bark treatments vs the control treatments (n=4 blanks for first experiment and n=7 sterilised bark treatments + n=3 blanks for the second experiment). We extracted genomic DNA from tree barks and sequenced the 16 rRNA universal marker gene for bacteria and archaea as well as the gene pmoA, specific of MOB. The 14 microbial extractions from bark also confirmed MOB abundance. The DFM MOB inhibitor experiments (n=88 trees) was compared to a control method using no DFM (n=39 trees) and this revealed significant differences between treatments (ANOVA on-ranks, $p < 0.001$).
Data collection	Nine bark samples from a common Australian wetland tree species (Melaleuca quinquenervia) were collected by LJ and placed into airtight bottles that were inoculated with methane. Ancillary parameters and measurements were recorded in notebooks. Using high precision CRDS, the isotopic enrichment of 13C and consumption of methane within each bottle and treatment were measured over time by LJ - indicative of MOB activity. These were data input directly into spreadsheet at the time of sampling. This experiment was repeated by LJ using 7 paired bark samples collected by LJ, that were also analyzed in duplicate (n=14) for microbial communities by EC, CG and PL, revealing unique MOB were markedly abundant in bark. The third investigation relied upon field based MOB inhibitor experiments conducted by LJ and DT on 88 trees. Stem flux rates were measured before the addition of DFM and after using a portable CRDS and stem flux chambers, with start and stop times recorded in notebooks and the positive % difference in fluxes indicative of bark dwelling MOB inhibition and in situ activity.
Timing and spatial scale	Using sterile methods, the bark for the first experiment was collected on the morning of the 4th of May 2020. The first inoculation time series experiment ran from the 4th - 12th of May 2020. The second (paired bark) samples were collected on the morning of the 20th of May 2020 and the second timeseries experiment ran until the 23rd of May 2020. The microbial samples and ancillary composite sediment and surface water samples were refrigerated within two hours of collection at 4 °C. They were later transported via courier to Monash University on 25th May 2020 and kept cold during transport and stored at -20°C until subsequent analyses. There was no strategic rationale for bark sample frequency, as the first experiment was to test our hypothesis that MOB may exist in bark via incubation isotope analysis, and the second experiment was instigated shortly afterwards in order to a) repeat the experiment successfully, whilst collecting additional bark swatches for microbial analysis, in order to confirm MOB communities and abundance. The DFM experiments were conducted over six days, during winter daylight hours between 29th June - 13th August 2020.
Data exclusions	In accordance with standard quality filtering approaches for raw sequences, low quality sequences were filtered out.
Reproducibility	Attempts to repeat the experiment were successful. Multiple samples showed similar results within each inoculation experiment. The DFM experiments were repeated over six times/ days. The inoculation experiment was performed twice with 9 successful replicates in the first experiment and then 7 successful replicates in the second. experiment
Randomization	The bark inoculation experiments consisted of three very simple treatments (one type of CH4 inoculation + bark), blanks (CH4 inoculation with no bark) and a sterile control (microwaved bark + CH4 inoculation). Allocation to experimental groups was not random, but rather based on sample collection location and the date of sampling, which was appropriate for this approach. The order of analysing the headspace in the inoculated bark treatments, microwaved sterile bark and blanks was randomized to avoid any potential artefacts within the CRDS equipment that may have influenced the results.
Blinding	The bark inoculation incubation experiment and microbial bark extractions were a quasi-paired blind experiment. SCU researchers conducted the isotopic bark incubation experiments, whilst Monash Univeristy researchers simultaneously analysed the bark microbial data, without knowing anything beyond the sample name nor having any idea of the results of the incubation experiments.

Only later were the two data streams (incubations and microbial communities) combined and correlations between MOB abundance and methane uptake revealed (Fig. 2c,d).

Did the study involve field work? ☒ Yes ☐ No

Field work, collection and transport

Field conditions	Heavy rains during February and March 2020 flooded the sampling location. Fieldwork was conducted during the Australian subtropical dry season of late Autumn and Winter of 2020, however the site was still inundated and the receding waters of the forest were in 0.5-2m of standing water during all sampling periods.
Location	-28.34982°, 153.57135°
Access & import/export	Permission was granted to sample the location from local land managers
Disturbance	Care was taken to minimise disturbances to the sampled trees and surrounding vegetation - as sterile methods were used to extract bark samples to avoid any cross-tree contamination. Small samples homogenized soil and water were collected for microbial analysis. The shallow soil sample holes were filled in to minimise disturbance.

Reporting for specific materials, systems and methods

We require information from authors about some types of materials, experimental systems and methods used in many studies. Here, indicate whether each material, system or method listed is relevant to your study. If you are not sure if a list item applies to your research, read the appropriate section before selecting a response.

Materials & experimental systems

n/a	Involved in the study
☒	☐ Antibodies
☒	☐ Eukaryotic cell lines
☒	☐ Palaeontology and archaeology
☒	☐ Animals and other organisms
☒	☐ Human research participants
☒	☐ Clinical data
☒	☐ Dual use research of concern

Methods

n/a	Involved in the study
☒	☐ ChIP-seq
☒	☐ Flow cytometry
☒	☐ MRI-based neuroimaging
